# Why promising ATMPs fail to reach patients: a qualitative stakeholder-informed analysis of cell therapy access in Europe

**DOI:** 10.1186/s12967-026-08303-x

**Published:** 2026-05-23

**Authors:** Giuseppe Di Mauro, Maria Pachetti, Christian Ploner, Michele De Luca, Graziella Pellegrini, Elisa Gambini, Massimo Moretti, Antonio Sfiligoj, Teodor E. Yordanov, Miomir Knezevic, Giulia Benedetta Sidoti, Giovanni Papa, Serena Zacchigna

**Affiliations:** 1 Cardiovascular Biology Laboratory, International Centre for Genetic Engineering and Biotechnology, Trieste, Italy; 2 Department of Plastic, Reconstructive and Aesthetic Surgery, Medical University of Innsbruck, Innsbruck, Austria; 3 Centre for Regenerative Medicine “Stefano Ferrari”, Department of Life Science, University of Modena and Reggio Emilia, Modena, Italy; 4 Vascular Biology and Regenerative Medicine Unit, Centro Cardiologico Monzino-IRCCS, Milan, Italy; 5 Oloker Therapeutics, Bari, Italy; 6 VivaBioCell S.p.A., Udine, Italy; 7 Angios FlexCo, Innsbruck, Austria; 8 GaiaCell, Advanced Cell and Gene Therapy Ltd., Trzin, Slovenia; 9 Plastic Surgery Department, Ospedale di Cattinara, Azienda Sanitaria Universitaria Giuliano Isontina, Trieste, Italy; 10 Department of Medicine, Surgery and Health Sciences, University of Trieste, Trieste, Italy

**Keywords:** ATMPs, HTA, Regulatory, Manufacturing, Funding, GMP, Scalability, Innovation, Tech transfer

## Abstract

**Background:**

Advanced therapy medicinal products (ATMPs) comprise gene therapies, somatic cell therapies, tissue engineered products (TEPs), and combined ATMPs. These modalities have the potential to deliver durable or curative benefit in oncological, genetic, chronic, and rare diseases with high unmet medical need. Yet in Europe, access remains limited, uneven, and often commercially unsustainable: clinically promising therapies frequently fail to progress beyond early development or are withdrawn after regulatory approval for reasons related to scalability and reimbursement rather than lack of clinical benefit.

**Methods:**

This study was designed as a qualitative, interview-based analysis to identify the major barriers faced by ATMP stakeholders to the clinical translation and patient access of cell therapies in Europe. We collected structured oral and written questionnaire/interview input from six stakeholders operating in the European ATMP landscape and synthesized the material into cross-cutting themes related to health technology assessment, manufacturing, regulatory implementation, and market dynamics.

**Results:**

Stakeholders consistently report that HTA approaches optimized for conventional pharmacological therapies often overemphasize short-term budget impact and insufficiently account for long-term clinical benefit, downstream healthcare and societal costs, and the durability of therapeutic effects. In parallel, substantial variability across donors, batches, manufacturing and delivery settings, together with fragmented GMP infrastructure, uneven national execution of clinical trial and access pathways, and chronic underinvestment, undermines reproducibility and scalability. Although the European Medicines Agency (EMA) offers facilitation instruments including early scientific dialogue and accelerated or supportive regulatory pathways, their potential to improve predictability is frequently attenuated by Member State heterogeneity and discontinuities across regional innovation ecosystems.

**Conclusion:**

From a stakeholder’s perspective, key recommendations including revising HTA frameworks to better reflect long-term lifetime value, investing to improve the accessibility to GMP facilities, strengthening ATMP regulatory expertise and access to early scientific advice, enabling cross-border ecosystems, and addressing structural market failure through public risk-sharing mechanisms.

## Introduction

ATMPs have progressed from a visionary concept to a concrete clinical reality, with a growing number of cell- and tissue-based approaches demonstrating meaningful, and sometimes durable, patient benefit. Yet the transition from laboratory innovation to routine patient access remains uniquely demanding. Unlike conventional pharmaceuticals, ATMPs are inherently complex and are affected by substantial variability across donors, batches, manufacturing conditions and delivery settings. As a result, translation is not only a scientific challenge, but also an industrial, regulatory, and health-economic one [1, 2].

**Fig. 1:**
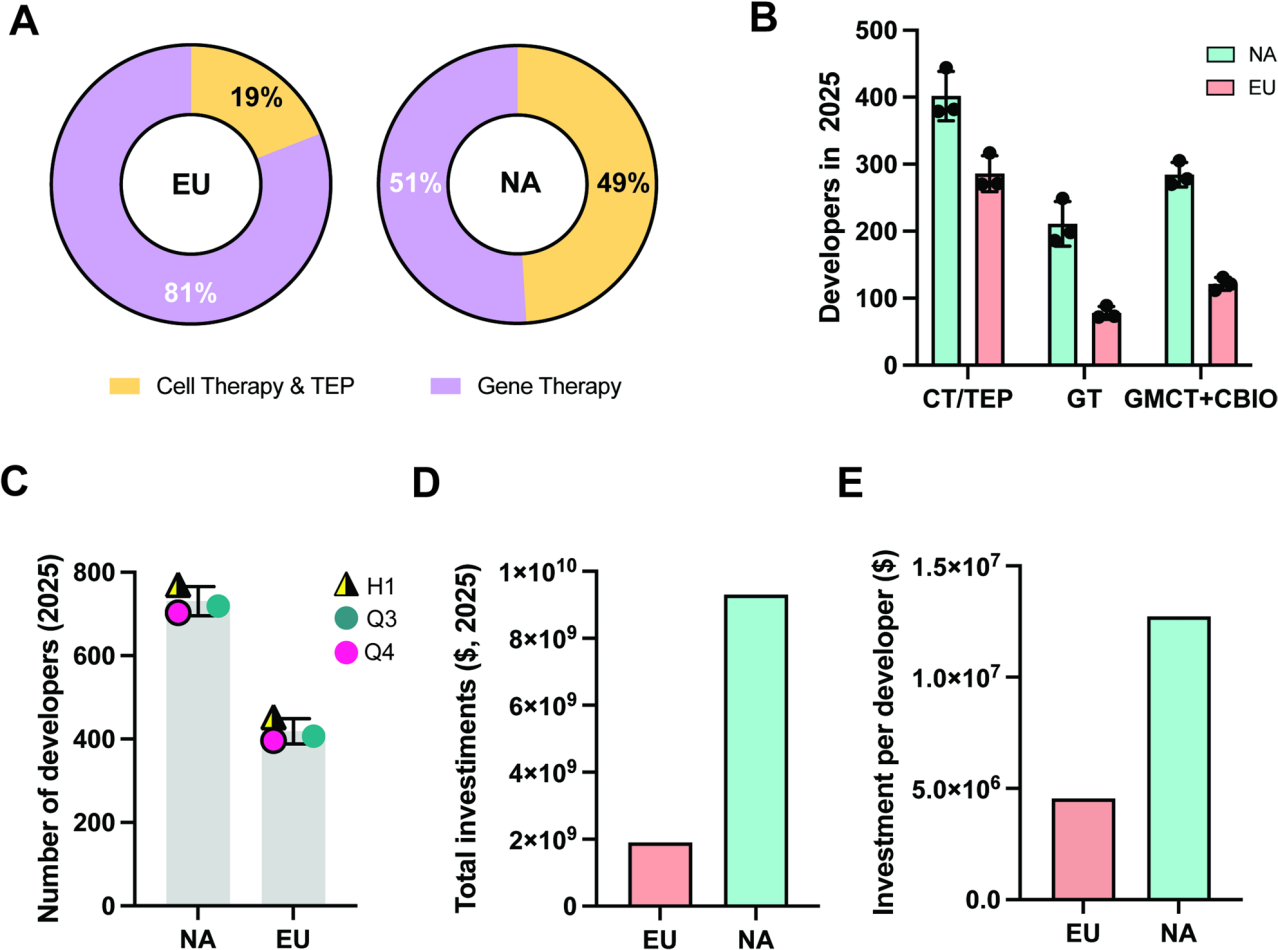
Approved ATMPs, developers and investments discrepancies in EU and NA regions. (**A**) Pie chart reporting the percentages of approved and still on the market cell therapies & TEP and gene therapies in EU and NA areas. (**B**) Cell therapy/TEP, gene therapy and genetically modified cell therapy + cell based immune-oncology (GMCT+CBIO) developers’ number trend in NA and EU in 2025. Dots represent the number of developers per categories in the H1, Q3 and Q4 period of 2025. (**C**) Total number of ATMPs developers in NA and EU in 2025. Dots represent the number of developers in the H1, Q3 and Q4 period of 2025. (**D**) Total investment in EU and NA in 2025; (**E**) average investment received by each ATMPs developer in EU and NA in 2025

To date, a pronounced imbalance exists between the number of approved gene and cell therapies, particularly in Europe, where gene therapy and cell therapy products account for more than 80% of authorized ATMPs, while cell therapies represent less than 20% (Fig. 1A) [3–6]. This imbalance is particularly notable given that cell therapy and tissue-engineered product (TEP) developers outnumber gene therapy developers in Europe, indicating a disconnect between the scale of innovation in the field and the number of therapies that ultimately achieve approval and patient access [7–9].

Gene therapies have predominantly been developed for rare, monogenic diseases, where the patient populations are relatively homogeneous and treatment benefit can be demonstrated with greater clarity. In contrast, many cell therapies target broader, multifactorial conditions; in these settings, demonstrating clinical efficacy typically requires larger and more complex trials, robust patient stratification, and the management of inherent variability in product potency and performance [1, 10]. That said, the present analysis focuses on why cell therapies, despite clinical promise, rarely reach routine patient use, particularly in the EU area. While some barriers affect the broader ATMP field, others are especially pronounced in cell therapies because of their intrinsic biological variability, more complex manufacturing workflows, and the difficulty of demonstrating reproducible efficacy across heterogeneous clinical settings [1, 10].

The structured interviews conducted with diverse stakeholders, including ATMP developers and biotechnology SMEs, provide a pragmatic perspective on the requirements and constraints associated with developing advanced cell therapies across different clinical contexts. Despite different technologies and indications, a set of recurring themes emerges such as scalability and reproducibility, regulatory challenges, clinical translation and patient accessibility. In this context, direct feedback from teams actively developing or having developed ATMPs is crucial to clarify which challenges have already emerged in practice and which ones may arise as the field scales. Beyond reporting achievements, these experiences highlight persistent system-level failures despite substantial advances at EU and Member State level and help translate lessons learned into practical guidance.

## Methods

This study was designed to qualitatively identify the major barriers faced by ATMP stakeholders in the clinical translation and patient access of cell therapies in Europe.

### Stakeholder selection

We engaged six stakeholders operating within the European ATMP landscape, including ATMP developers, biotechnology small and medium-sized enterprises (SMEs) and translational leaders. The selected stakeholders represented complementary experience across product development, GMP manufacturing, regulatory navigation, clinical translation, and market access. The experts were identified through cross-border translational initiatives established to improve patient access to a cell therapy. They are mainly based in Italy, Austria and Slovenia. One contributor requested anonymity and is therefore reported in anonymized form. Stakeholders and their main domains of expertise are summarized in Table 1.

### Data collection and synthesis

The questionnaire/interviews took place between September 2025 and February 2026. The interviews lasted approximately 1 h. Both interviews and written questionnaires were organized around five core domains: *i*) experience in developing the cell therapy product, including its characteristics and target indications; *ii*) major challenges encountered in translating the approach to patients; *iii*) strategies adopted to address these challenges; *iv*) anticipated future challenges; *v*) future plans and strategic objectives. Data were collected and organized into a structured set of responses. These responses were subsequently reviewed with the stakeholders in order to correct or clarify them.

This structure was chosen to capture both retrospective and forward-looking perspectives, while allowing comparison across stakeholders operating in different therapeutic and organizational settings. The collected material was reviewed and consolidated into thematic areas with the aim of identifying recurrent barriers, enabling strategies, and priorities. Representative stakeholder examples were retained to illustrate convergence points.

## Results

Structured interviews with ATMP developers, SMEs, and stakeholders revealed convergent experiences across different technological, clinical, and organizational settings (Table 1). Although the products and target indications varied substantially, the same bottlenecks repeatedly emerged. Stakeholder perspectives converged around four major themes: misalignment of HTA and reimbursement systems, manufacturing and GMP constraints, regulatory fragmentation, financing and ecosystem-level challenges. These barriers varied according to the maturity of the product, disease area, and organizational model of each stakeholder.

### HTA and reimbursement systems remain poorly aligned with long-term therapeutic value

A recurring message across stakeholders was that current HTA and reimbursement frameworks remain inadequately adapted to cell therapies, especially when these therapies involve high upfront costs but may provide durable or potentially curative benefit. Stakeholders consistently described a misalignment between the economic logic of current systems and the long-term value profile of advanced therapies, particularly in rare, chronic, or highly disabling conditions.

*Holostem Terapie Avanzate* emphasized that ATMPs are still frequently evaluated through short-term cost metrics derived from conventional pharmaceuticals, even though some of these therapies may fundamentally alter the patient’s long-term clinical trajectory. Their perspective highlighted the contrast between the high apparent cost of a one-time therapy and the chronic cumulative cost of leaving severe disease untreated. *EduCell/GaiaCell* likewise stressed that personalized and autologous cell therapies do not fit the conventional pharmaceutical business and reimbursement models, thereby making commercialization difficult even when the clinical rationale is strong. *Oloker Therapeutics* also emphasized that reimbursement and health-economic evaluation are likely to become decisive barriers in cardiovascular indications, where wider adoption beyond highly specialized centers will depend on robust comparative evidence and appropriate recognition of long-term value.

Taken together, these perspectives suggest that one of the main barriers to patient access is not only regulatory approval, but the inability of current assessment and reimbursement systems to appropriately capture the lifetime value of many cell therapies.

### Manufacturing, GMP access, and scalability remain central bottlenecks

Manufacturing-related constraints emerged as one of the most consistent themes across interviews. Stakeholders repeatedly described cell-based products as intrinsically sensitive to donor variability, batch effects, process conditions, and delivery settings. These features make reproducibility, scale-up, potency assessment, and quality control particularly demanding compared with more conventional therapeutic products.

*Holostem Terapie Avanzate* emphasized that, despite the existence of technologies for automation and industrialization, production has often remained largely manual because of insufficient investment from both public and private actors. They also highlighted the major burden imposed by repeated batch-specific quality controls for living cell products. *Oloker Therapeutics* described the challenge of designing a process that was not only GMP-compliant but also scalable and compatible with the operational constraints of the selected cell factory, while ensuring potency consistency across batches. *Angios FlexCo* identified the transition from academic protocols to GMP-compliant manufacturing as its main current translational bottleneck. The *anonymous* stakeholder similarly reported major difficulties in preserving myoblast viability, purity, and regenerative potential during large-scale GMP-compliant expansion and delivery. *VivaBioCell* emphasized automation and bioreactor-based approaches as routes to improve reproducibility and cost control, while *EduCell/GaiaCell* highlighted decentralized manufacturing as a potentially more flexible implementation strategy in European contexts.

Overall, stakeholders did not report the scalability as impossible, but as dependent on broader access to infrastructure, process optimization, and sustained investment.

### Regulatory support exists, but implementation remains fragmented across Europe

The third major theme was the fragmentation of regulatory and institutional implementation across Member States. Stakeholders recognized the value of facilitation tools at EU level, including scientific advice and other supportive interactions with regulators. However, they also stressed that the practical value of these instruments is often weakened by heterogeneous national procedures, variable institutional readiness, and inconsistent pathways for ethics review, trial activation, and clinical implementation.

*Holostem Terapie Avanzate* described a long historical trajectory in which early legal and regulatory frameworks were poorly adapted to living, complex, and personalized products. *EduCell/GaiaCell* reported that, once advanced therapy legislation was introduced, implementation still varied markedly across Member States, making international navigation especially difficult for SMEs from smaller countries. *VivaBioCell* highlighted the lack of ATMP-specific procedures within regional ethics systems and the difficulty of obtaining even preliminary institutional commitment in some settings. By contrast, *Oloker Therapeutics* pointed to the value of closer interaction with regulators and described proactive engagement with competent authorities and scientific advice procedures as an important way to anticipate obstacles and align development plans earlier.

Such evidence suggests that the main issue is not simply regulatory stringency, but uneven implementation and limited continuity across the European translational pathway.

### Financing models and ecosystem structures remain poorly adapted to cell therapies

Financing and business sustainability were repeatedly identified as structural barriers. Across interviews, stakeholders described a persistent mismatch between the development profile of cell therapies and the expectations of traditional private investment models. Long development timelines, small or heterogeneous patient populations, uncertain reimbursement, and delayed return on investment were all described as factors that weaken investor confidence and limit commercial sustainability.

*Angios FlexCo* reported that fundraising is currently a prerequisite for further progress toward GMP adaptation and clinical development. *EduCell/GaiaCell* emphasized the difficulty of crossing the translational “valley of death,” especially once projects move beyond early-stage grants and require substantial capital for clinical trials and regulatory advancement. The *anonymous* stakeholder also stressed that private capital has become increasingly difficult to secure because many cell therapy projects fail to deliver returns on a time scale that is attractive to short-term investors. *Holostem Terapie Avanzate* described the importance of combining heterogeneous funding sources and multidisciplinary collaborations. *VivaBioCell* emphasized that a cross-border ecosystem model involving hospitals, universities, biotech companies, innovation hubs, and investors enabled a more integrated approach to translation than a purely linear product-development model.

Across stakeholders, a recurring view was that successful translation depends not only on the product itself, but on the existence of coordinated systems that connect manufacturing, regulation, clinical expertise, and financing.

**Table 1:** Stakeholders interviewed and their domain of expertise, and cell therapy context

Interview/ Organization	Type of stakeholder	Main domain of expertise	Cell therapy target
**Holostem Terapie Avanzate** - Prof. Michele De Luca (co-founder/CSO) and Prof. Graziella Pellegrini (co-founder/scientific expert and R&D manager)	Academic/clinician-scientist; Scientific expert, pioneer of EMA-authorized ATMPs	ATMP development; GMP manufacturing; clinical trials; regulatory pathways; market authorization	Authorized epithelia stem cell therapies, including Holoclar, as well as other epithelial and gene therapy application
**Oloker Therapeutics** - Dr. Elisa Gambini (COO-CTO)	SME	Early-stage ATMP development; GMP manufacturing; SME regulatory navigation	Cardiac progenitor cell-based ATMP for refractory angina and other cardiovascular indications
**Angios FlexCo –** Dr. Teodor E. Yordanov (CSO)	SME/Executive and translational leadership	Development of advanced cell-based therapies; scale-up strategies; interaction with regulatory and reimbursement frameworks	Organoid-based vascular platform for wound healing and tissue regeneration
**EduCell; GaiaCell Advanced Cell and Gene Therapy -** Dr. Miomir Knezevic (Scientific Director)	SME	Autologous and allogeneic cell therapy development; HE framework; orthopedic and immunological ATMPs; decentralized manufacturing models	Autologous and allogenic cell therapies including cartilage repair, GvHD, CAR-T and TIL-based approaches
**VivaBioCell** - Dr. Antonio Sfiligoj (CEO) and Dr. Massimo Moretti (Lab Manager)	SME/Coordination and industrial leadership	EU-funded translational programs; shared GMP manufacturing platforms; public-private collaboration	Adipose-derived stem cell applications and automated bioreactor-based manufacturing
**Anonymous**: Senior Technical Operations	SME	Development of ATMPs	Myoblast-based regenerative platform targeting anal and bladder sphincter muscle loss

## Discussion

A substantial gap exists between approved gene- and cell-therapy medicinal products, particularly in Europe. Figure 1A illustrates the portion of gene- and cell therapies approved and currently on the market in the EU and North America (NA) areas, respectively [3–6]. In the EU, approved gene therapies outnumber cell therapies approximately four-fold, while in NA the disparity is less pronounced. Interestingly, in NA, the ratio of developers working on cell therapy and TEP relative to those focused on gene therapy is approximately 1.9, indicating that the cell/TEP segment is nearly twice the size of the gene therapy segment (Fig. 1B) [7–9]. In Europe, this ratio increases to 3.7, highlighting an even more pronounced imbalance: cell therapy and TEP developers substantially outnumber gene therapy developers [7–9].

Despite the total number of ATMP developers in the EU being 1.7-fold lower than in NA (Fig. 1C), this does not translate into a higher average funding per developer. On the contrary, the overall scale of ATMP investment in 2025 is about 4.9-fold higher in NA than in the EU, further widening the gap both in development capacity and in the speed of industrial translation (Fig. 1D). Within this funding landscape, the average funding per developer in the EU is consequently 2.8-fold lower than in NA (Fig. 1E). That said, one plausible reason for the more pronounced gap is that the EU hosts a relatively larger number of cell-therapy and TEP developers competing for substantially less capital, which can translate into slower clinical progression, more constrained manufacturing scale-up, and fewer resources for late-stage development and commercialization. These quantitative discrepancies suggest that the limited number of approved cell therapies in Europe is unlikely to reflect a lack of scientific activity alone, but rather downstream translational bottlenecks along the path from development to patient access.

This situation is further constrained by the current regulatory landscape in the EU. While the European regulatory framework for ATMPs is robust, it is operationally complex. ATMPs are governed by a largely ATMP-specific body of regulatory guidance with limited harmonization across other biological product categories [11]. The European Medicines Agency (EMA), in coordination with the International Council for Harmonisation of Technical Requirements for Pharmaceuticals for Humane use (ICH), plays a central role in structuring this regulatory landscape. The EMA provides facilitation instruments to support developers and reduce regulatory uncertainty, including early scientific engagement mechanisms such as Innovation Task Force briefings and formal scientific advice, support programs for selected products and developers including the PRIortiy MEdicines (PRIME) scheme and pilot initiatives for academic and non-profit developers, and regulatory incentives for rare diseases through orphan designation. Evidence suggests that these mechanisms can improve regulatory predictability and accelerate approval timelines for products that qualify for facilitated pathways [12]. However, stakeholder interviews constantly indicate that the critical bottlenecks arise downstream of EU-level regulatory support, at the level of national implementation and healthcare system preparedness (Table 2). Although marketing authorization is centralized, clinical trial authorization and execution remain largely under the authority of national competent agencies and ethics committees. ATMP developers report that country-specific procedures and uneven institutional readiness contribute to delays during clinical translation, often before EMA-level milestones are reached. Stakeholders emphasize that early and proactive engagement with regulatory authorities is essential: initiating dialogue at early stages of development enables alignment of manufacturing, preclinical and clinical strategies with regulatory expectations, thereby reducing the risk of costly redesign, minimizing delays and improving adherence to investor-agreed business plan timelines. A comparison with non-EU regulatory environments helps contextualize these challenges. In the US, regenerative medicine products may access dedicated expedited interactions through RMAT designation within the FDA framework [13], while in Japan innovative products may benefit from nationally administered acceleration mechanisms such as the SAKIGAKE designation system and regenerative medical product pathways [14]. The European framework also offers important support tools, including PRIME and scientific advice, but the stakeholder perspectives collected here suggest that their practical impact is more often attenuated downstream, at the level of national implementation, ethics procedures, and healthcare-system readiness. The relevant contrast, therefore, is not the absence versus presence of fragmentation, but the degree of continuity linking early regulatory support to later-stage clinical implementation and patient access.

Academic institutions are a key driver of ATMP development. Interestingly, approximately 25% of currently approved ATMPs in the EU originate from academic settings [15]. However, academic groups typically have limited expertise in regulatory strategy, regulatory practices, clinical development, intellectual property management, business development and commercialization processes [1]. One stakeholder interview identified this translational capability gap as a significant barrier to broader clinical implementation, particularly for cell therapies. In addition to regulatory and operational constraints, academic developers often face structural limitations in accessing private investment, relying predominantly on public research funding. This funding model incentivizes scientific output but does not adequately support the downstream activities required for clinical translation, regulatory approval, and market access. Despite limited preparedness in regulatory, intellectual property, and marketing matters, academics are less prone to secure private funding, while relying on public grants for their research, while being constrained by the “publish or perish” logic of academic careers. To address these barriers, several European technology transfer and translational initiatives have been established to facilitate the conversion of academic discoveries into clinically deployable therapies [2, 16]. Accordingly, EMA and European University Hospital Alliance (EUHA) launched initiatives focused on building multi-stakeholder ecosystems that bring together complementary expertise across academic centers, hospitals, manufacturing platforms, regulators and evaluators, with the aim of making the development, approval and assessment of cell therapies as fluid as possible [17, 18].

A growing body of evidence indicates that translating scientific discoveries into clinical and social solutions benefits from collaboration among academic researchers, clinicians, industry partners, regulatory experts, and investors [19]. An example of such an integrated approach is the Advance Therapies model developed by Lund, which establishes a coordinated framework linking universities, hospitals and specialized translational actors, including regulatory experts, intellectual properties experts and incubators [20]. This model preserves the functional independence of each stakeholder while maintaining a comprehensive overview of the project. The need for integrated and coherent cooperation among stakeholders (academia and SMEs, regulatory bodies, investors, and infrastructure providers) clearly emerged from our interviews, highlighting both the urgency to act and the need for shared translational language and training across sectors. These translational limitations become even more evident when academic discoveries move toward GMP adaptation, industrialization, and clinical implementation, where manufacturing robustness and infrastructure availability become decisive.

Manufacturing remains a critical bottleneck in translating ATMPs from clinical development to routine patient care. Many ATMPs still rely on small-batch production, intensive quality control, and high per-patient costs, with scalability limited by process sensitivity and the need to maintain functional performance across production runs. While enabling solutions such as automation, closed-system workflows, and modular GMP platforms are increasingly available, their implementation requires coordinated investment and regulatory alignment, and access to dedicated infrastructure remains uneven [10]. Unlike conventional pharmaceuticals, which are produced as uniform products at scale for broad patient populations, ATMPs often require ad hoc, patient-specific production and business models [21]. The substantial costs of clinical trials further contribute to the high overall investment needed to bring ATMPs to market [1]. Stakeholder interviews highlight that manufacturing is not only a downstream operational step but a critical determinant of translational feasibility, shaping reproducibility, scalability, trainings needs, cost, and timelines. Participants emphasize the tension between GMP compliance and economic sustainability: while robust process control, stringent in-process monitoring, and clear release criteria are clearly defined to assure quality and safety, many ATMP workflows remain effectively artisanal due to underfunded industrialization and limited structural support for automation. A recurring technical challenge is maintaining functional performance and batch-to-batch consistency during scale-up and tech transfer to cell factories, particularly for products affected by intrinsic variability or facility-specific constraints such as capacity, closed-system compatibility, scheduling, and operational limits. Product-specific fragility such as loss of purity during expansion, reduced viability, and inadequate final yields, underscores the importance of process optimization, sometimes elevating delivery-enabling technologies to a core component of reproducible clinical performance. Key strategies to address these challenges include progressive adoption of closed systems, strengthened in-process controls, and, critically, automation and modular or decentralized manufacturing platforms including “GMP-in-a-box” concepts. These approaches reduce operator-dependent variability, improve standardization, and contain costs. Overall, the interviews suggest that scalability is constrained less by the absence of technical solutions than by uneven access to ATMP-dedicated GMP infrastructure, limited industrial partnerships, and discontinuous investment. An important distinction emerging from both the stakeholder interviews and the literature is that some barriers are broadly shared across the ATMP field, whereas others are especially pronounced in cell therapies. Cross-cutting ATMP-wide barriers include HTA misalignment, regulatory fragmentation across Member States, and weak financing models for advanced therapies [20–24]. By contrast, challenges that are particularly prominent in cell therapies include donor and batch variability, sensitivity to process conditions, complex potency assessment, loss of viability or purity during expansion, and scale-up difficulties for living products, all of which complicate reproducible manufacturing and technology transfer [1, 10]. Clarifying this distinction is important, because improving access will require both general ATMP reforms and solutions tailored to the biological and manufacturing specificities of cell-based products. However, even when regulatory and manufacturing barriers can be partially addressed, long-term sustainability ultimately depends on whether these therapies can be appropriately valued and reimbursed within national healthcare systems.

A major barrier to ATMP adoption in Europe lies in the evaluation of their value within national healthcare systems. ATMPs are often assessed through HTA frameworks originally developed for conventional pharmacological therapies. These frameworks tend to emphasize short-term budget impact, especially when long-term data are lacking, as well as upfront acquisition costs, which can underrepresent the value of one-time or long-lasting interventions capable of delivering durable clinical benefit [21]. This methodological mismatch frequently results in delayed reimbursement decisions, restricted indications, and fragmented patient access, even when long-term cost-effectiveness is proven or plausible because downstream savings, such as reduced hospitalizations, avoidance of long-term disability, lower supportive care requirements and productivity gains are insufficiently weighted [22, 23]. Moreover, effective pricing strategy must balance incentivizing innovation with ensuring value-for-money for payers, assigning a fair price to new health technologies [24].

Stakeholders consistently advocate for a fundamentally different cost-benefit framework for ATMPs. Evaluation should consider the full longitudinal cost of disease, including chronic care utilization, disability, loss of productivity, caregiver burden, and downstream complications, over an appropriate lifetime horizon. In this perspective, the relevant comparator is not the short-term unit price of a single treatment, but the extent to which the therapy modifies a patient’s clinical trajectory over decades. The issue is further compounded for rare and ultra-rare diseases, where the high per-patient cost of ATMP can lead to prolonged negotiations or outright rejection from the national health systems, despite treatment efficacy. Due to this situation, commercial pressure may even lead companies to withdraw an approved and effective ATMP for marketing reasons [1].

Without modernization of HTA methodologies and reimbursement policies, uncertainty regarding coverage and price sustainability will continue to slow patient access and discourage long-term investment and innovation in advanced therapies, including related satellite companies. This is particularly critical for rare, ultra-rare, and heterogeneous conditions where conventional evidence and payment paradigms are poorly aligned with the characteristics of ATMP [24]. Taken together, these observations indicate that the bottlenecks emerging from our stakeholder interviews are consistent with barriers already highlighted in the broader literature, suggesting that they reflect recurrent structural weaknesses in the European translation of cell therapies rather than isolated local concerns.

### Limitations of the present study

This analysis is based on a small number of stakeholders and is therefore not intended to provide a statistically representative assessment of the European ATMP sector. The sample is geographically concentrated in Europe (Italy, Slovenia, Austria) and reflects primarily the perspectives of developers, SMEs, and translational actors involved in cell therapy and regenerative medicine, rather than the full spectrum of stakeholders relevant to ATMP access, such as payers, large pharmaceutical companies, or national regulators. In addition, stakeholder selection may introduce perspective bias, as participants were purposively included based on their direct experience in the field. Some contributors also hold professional roles in companies active in the sector, which may influence how challenges and priorities are framed, although these competing interests are explicitly disclosed. Finally, while several barriers identified here are relevant across the broader ATMP field, the present analysis focuses mainly on cell therapies and should therefore not be generalized to all ATMP categories or all EU Member States.

**Table 2:** Main messages derived from interviews

Interview/ Organization	Key challenges	How they tackle those challenges	Future challenges and objectives
Holostem Terapie Avanzate	ATMP development in absence of ATMP regulatory framework; lack of ATMP manufacturing scalability; low investments on industrialization; ATMP evaluation with unproper HTA metrics.	Establishment of public-private network for investments; early-dialogue with regulatory offices.	Establishing multidisciplinary teams to tackle challenges faced by ATMPs; Automation; Decentralization of production.
Oloker Therapeutics	Finding proper industrial partners; scalability and efficacy in GMP-contexts; complex regulatory frameworks.	Establishment of specialized partners’ network; optimization of manufacturing in close and automated systems; early contact with regulatory offices; consistent investments in predictive in large pre-clinical models.	Demonstrate durability of the therapy benefits; scalability/costs per patient; adoption in the cardiovascular field; HTA and reimbursement.
Angios FlexCo	Challenges in aligning clinical development with manufacturing scalability; need for predictable regulatory timelines; impact of reimbursement uncertainty; need for big investments.	Secure enough funding to scale up and translate pre-clinical results.	Increasing importance of animal-free in vitro approaches through organoids; regulatory framework should embrace an increasingly complex class of biologics. Engage strategic partners capable of supporting the transition from preclinical validation to clinical application.
EduCell/GaiaCell	Regulatory fragmentation across Member States; misalignment with blockbuster pharma model; funding “valley of death”; manufacturing standardization challenges.	Sustainable organic growth; academic-clinical partnerships; grant diversification; decentralized production models; HE pathway.	Automation and cost optimization; development of potency assays; cell-derived products (EVs); improved reimbursement models; international partnerships.
VivaBioCell	Absence of public and political support; misusing of GMP facility; low institutional and ethics committee readiness.	Creation of a cross-border ecosystem, incorporating different expertise; adoption of an automated device for the manufacturing process.	Emergence of under-funded SMEs across EU and of competitive regulatory frameworks in other regions (e.g. China or Japan); emergence of cell-free approaches. Need for EU industrial partner to scale up; collaboration between academia/SMEs/big pharma company in EU and US.

## Conclusions

The stakeholder perspectives collected in this study suggest that, for cell therapies in Europe, the main bottleneck is not the scientific feasibility of the therapies themselves, but the misalignment of the systems required to bring them to patients. In particular, gaps between regulatory implementation, manufacturing capacity, and therapy value assessment continue to constrain routine clinical access. While the EMA provides facilitation instruments that can materially improve predictability, such as early dialogue and accelerated or supportive pathways, their impact is often attenuated by persistent fragmentation at national level. Country-specific procedures, uneven ethics and institutional readiness, discontinuous GMP infrastructure planning, and insufficient investment contribute to heterogeneous timelines and geographic disparities in access. Taken together, the stakeholders’ experiences indicate that improving access to cell therapies will require closer alignment across governance, industrialization, and health-system incentives to support the inherently complex, resource-intensive and time-consuming development of ATMPs. This includes revising HTA and reimbursement frameworks to capture lifetime benefit, durable outcomes, and avoided downstream healthcare and societal costs; expanding access to GMP capacity through government-supported or academic, shared, decentralized or modular platforms; and strengthening regulatory expertise together with early scientific dialogue for developers.

Overall, this qualitative stakeholder-informed analysis supports the view that Europe already possesses much of the scientific capability and regulatory foundation needed to lead in advanced therapies, but that translation remains constrained by fragmentation in implementation and investment. Although the present study is based on a limited number of stakeholder interviews and is not intended to provide a representative assessment of the entire ATMP field, the bottlenecks identified here are consistent with challenges already highlighted in the broader literature, suggesting that they reflect recurrent structural weaknesses in the European translation of cell therapies rather than isolated stakeholder-specific concerns. Improving durable patient access will therefore depend on moving from fragmented, case-by-case pathways toward a more integrated ecosystem approach, particularly for the biological, manufacturing, and market-access challenges that are especially pronounced in cell therapies.

## Data Availability

The dataset used in the current study is reported in https://alliancerm.org/resources/?_resource_type=cell-gene-therapy-sector-data.

## Abbreviations

ATMP: Advanced Therapy Medicinal Product
TEP: Tissue Engineered Products
HTA: Health Technology Assessment
SME: Small-Medium Enterprises
EMA: European Medicines Agency
EUHA: European University Hospital Alliance
GMP: Good Manufacturing Practices
CSO: Chief Scientific Officer
COO: Chief Operating Officer
CTO: Chief Technology Officer
CEO: Chief Executive Officer
MAA: Marketing Authorization Application
iPSCs: Induced Pluripotent Stem Cells
BVO: Blood Vessels Organoid
CPCPlus: Cardiac Pro-angiogenic Cell Plus
ACI: Autologous Chondrocyte Implantation
MSC: Mesenchymal stem cells
GvHD: Graft-versus-Host Disease
ARDS: Acute Respiratory Distress Syndrome
TIL: Tumour-infiltrating Lymphocytes
ADSC: Adipose-tissue Derived Stem Cell
QC: Quality Control
NHS: National Health System
JAZMP: Javna Agencija Republike Slovenjie za zdravila in medicinske pripomočke
FDA-IND: Food and Drug Administration Investigational New Drug
ROI: Return On Investment
IPO: Initial Public Offering
NA: North America
EU: European Union, CT: Cell Therapy
GT: Gene Therapy
CBIO: Cell-Based Immune-oncology
GMCT: Genetically Modified Cell Therapy
ICH: International Council for Harmonization of Technical Requirements for Pharmaceuticals for Human use
PRIME: Priority Medicine
RMAT: Regenerative Medicine Advanced Therapy
EV: Extracellular vesicles
